# What’s on a prophage: analysis of *Salmonella* spp. prophages identifies a diverse range of cargo with multiple virulence- and metabolism-associated functions

**DOI:** 10.1128/msphere.00031-24

**Published:** 2024-05-22

**Authors:** Caroline R. Yates, Anthony Nguyen, Jingqiu Liao, Rachel A. Cheng

**Affiliations:** 1 Department of Food Science and Technology, Virginia Tech, Blacksburg, Virginia, USA; 2 Computational Modeling and Data Analytics Program, Virginia Tech, Blacksburg, Virginia, USA; 3 Department of Civil and Environmental Engineering, Virginia Tech, Blacksburg, Virginia, USA; U.S. Food and Drug Administration, Silver Spring, Maryland, USA

**Keywords:** *Salmonella*, prophage, bacteriophage, antimicrobial resistance genes

## Abstract

The gain of mobile elements, such as prophages, can introduce cargo to the recipient bacterium that could facilitate its persistence in or expansion to a new environment, such as a host. While previous studies have focused on identifying and characterizing the genetic diversity of prophages, analyses characterizing the cargo that prophages carry have not been extensively explored. We characterized prophage regions from 303 *Salmonella* spp. genomes (representing 254 unique serovars) to assess the distribution of prophages in diverse *Salmonella*. On average, prophages accounted for 3.7% (0.1%–8.8%) of the total genomic content of each isolate. Prophage regions annotated as Gifsy 1 and Salmon Fels 1 were the most commonly identified intact prophages, suggesting that they are common throughout the *Salmonella* genus. Among 21,687 total coding sequences (CDSs) from intact prophage regions in subsp. *enterica* genomes, 7.5% (median; range: 1.1%–47.6%) were categorized as having a function not related to prophage integration or phage structure, some of which could potentially provide a functional attribute to the host *Salmonella* cell. These predicted functions could be broadly categorized into CDSs involved in: (i) modification of cell surface structures (i.e., glycosyltransferases); (ii) modulation of host responses (e.g., SodC/SodA, SopE, ArtAB, and typhoid toxin); (iii) conferring resistance to heavy metals and antimicrobials; (iv) metabolism of carbohydrates, amino acids, and nucleotides; and (v) DNA replication, repair, and regulation. Overall, our systematic analysis of prophage cargo highlights a broader role for prophage cargo in influencing the metabolic, virulence, and resistance characteristics of *Salmonella*.

**IMPORTANCE:**

Lysogenic bacteriophages (phages) can integrate their genome into a bacterial host's genome, potentially introducing genetic elements that can affect the fitness of the host bacterium. The functions of prophage-encoded genes are important to understand as these genes could be mobilized and transferred to a new host. Using a large genomic dataset representing >300 isolates from all known subspecies and species of *Salmonella*, our study contributes important new findings on the distribution of prophages and the types of cargo that diverse *Salmonella* prophages carry. We identified a number of coding sequences (CDSs) annotated as having cell surface-modifying attributes, suggesting that prophages may have played an important role in shaping *Salmonella*'s diverse surface antigen repertoire. Furthermore, our characterization of prophages suggests that they play a broader role in facilitating the acquisition and transfer of CDSs associated with metabolism, DNA replication and repair, virulence factors, and to a lesser extent, antimicrobial resistance.

## INTRODUCTION

*Salmonella* is a diverse foodborne pathogen that accounts for over 90 million illnesses globally each year (1, 2). Within the genus *Salmonella*, there are two species, *Salmonella bongori* and *Salmonella enterica*; *S. enterica* can be divided further into six recognized subspecies *enterica* (I), *salamae* (II), *arizonae* (IIIa), *diarizonae* (IIIb), *houtenae* (IV), and *indica* (VI), and an additional proposed subspecies *londinensis* (VII) (3). *Salmonella enterica* subsp. *enterica* can be further categorized into four major phylogenetic clades (A–D); this classification has been useful for understanding the distribution of horizontally acquired elements among diverse *Salmonella* serovars (4). *Salmonella*’s lipopolysaccharide (LPS) and flagellar structures constitute the O and H antigens, respectively, that are the basis for the Kauffman–White–Le Minor serotype classification scheme that remains useful for surveillance. There are over 2,600 recognized serovars, with 1,586 belonging to *S. enterica* subsp. *enterica* (5). Importantly, some serovars may evolve to express the same serotype despite evolving from different most recent common ancestors, leading to so-called polyphyletic serovars (4).

*S. enterica* is associated with a wide variety of hosts and environments, which reflects the genetic diversity of this pathogen (6). Genes that have important relevance to human disease include those whose gene products facilitate (i) enhanced survival under physiological stress such as the environment encountered in the host gastrointestinal tract, (ii) utilization of host-generated metabolites, and (iii) resistance to antimicrobial treatments, all of which are known to vary by serovar (7, 8). Multiple studies have demonstrated that virulence potential can vary by serovar and strain, highlighting the limitations of current serotype-dependent surveillance frameworks, as well as the importance of understanding the genetic diversity of *Salmonella* (9, 10).

Bacteriophages (viruses that infect bacterial hosts) are the most abundant organisms in nature, having an estimated population of 10^31^ members (11). Temperate bacteriophages are capable of undergoing both lytic and lysogenic life cycles. During the lysogenic phase, bacteriophages are able to integrate their genome into the host bacterium’s genome, forming a prophage (11). When the bacterial host experiences physiological stress (e.g., DNA damage, excessively high temperatures, antimicrobial treatment), prophage induction can occur, causing the bacteriophage to enter the lytic cycle producing virions that ultimately lyse and kill the host cell (12). Depending on the prophage, these genomic regions may be maintained in the genome (i.e., remain “intact”), or they may be degraded (leading to cryptic prophages). In addition to encoding genes necessary for their own replication and assembly, prophages are also important facilitators of horizontal gene transfer, often carrying CDSs that have been acquired from prior hosts as their “cargo” (13).

*S. enterica* is known to encode a variety of prophages, including Gifsy 1 and Salmon Fels 2 (4, 14). Some studies have demonstrated a role for prophages in facilitating acquisition of virulence factors, such as SodC, which has been shown previously to enhance *Salmonella* survival during macrophage infection (15, 16). Although previous work by Brussow et al. (17) has indicated that prophages likely provide some benefit to their bacterial host, the specific types of cargo that prophages carry, and the potential functional benefit of these cargo, remain poorly understood. Therefore, we aimed to (i) systematically characterize the diversity and distribution of prophages across seven *S*. *enterica* subspecies and *S. bongori* to contextualize the distribution of prophages in genetically diverse *Salmonella* spp. isolates, and (ii) assess the predicted functions of CDSs within intact prophage regions to understand what potential benefits these CDSs confer to lysogenized *Salmonella*.

## RESULTS

### *S. enterica* subsp. *enterica* genomes encode diverse prophages, which account for an average of 4% of the total genomic content

**Fig 1:**
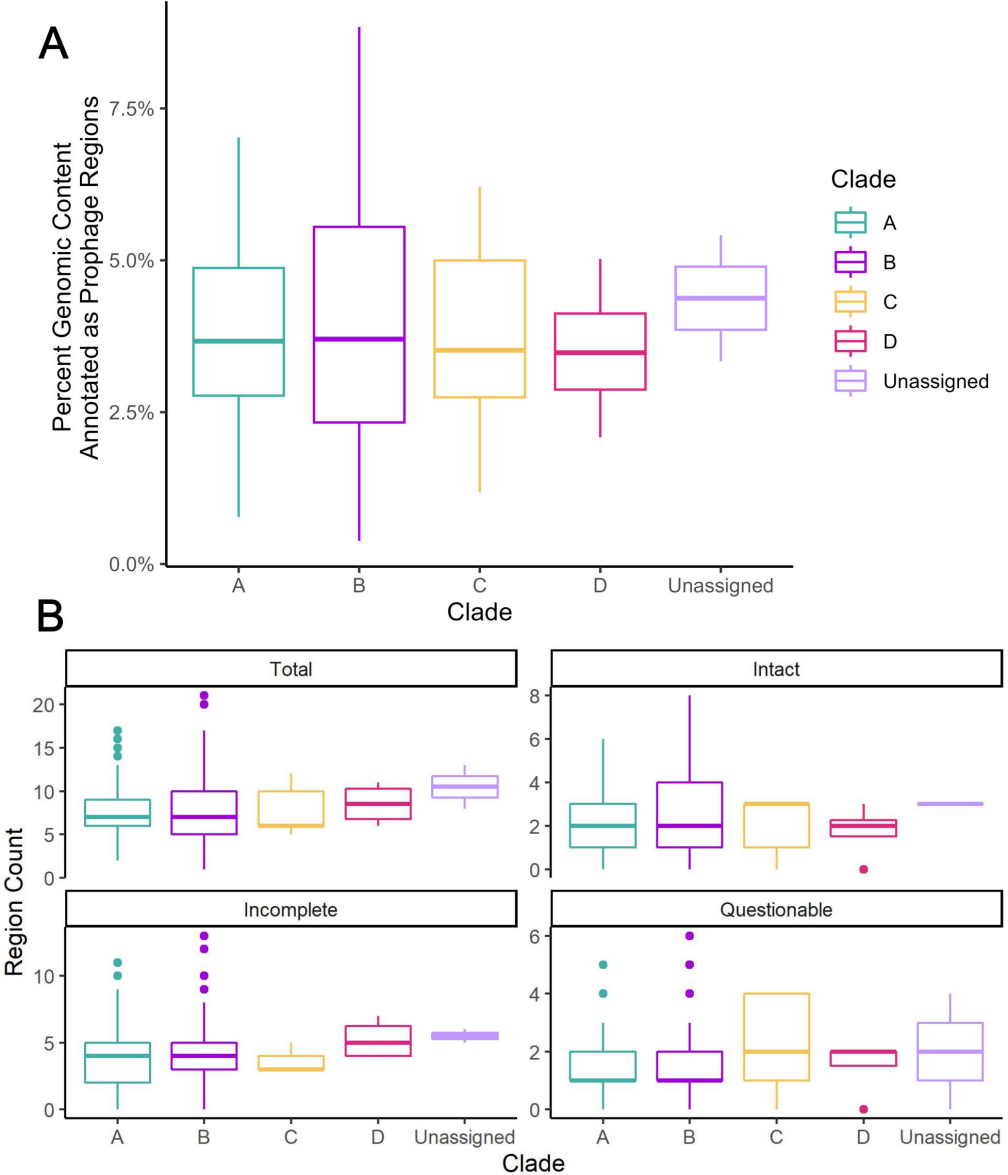
Distribution of prophage regions among 242 *S*. *enterica* subsp. *enterica* genomes representing 217 unique serovars and 25 poly or paraphyletic serovars. Prophage regions within the 242 *S. enterica* genomes (clade A [*n* = 152 genomes], clade B [*n* = 79 genomes], clade C [*n* = 5 genomes)], clade D [*n* = 4 genomes], and clade Unassigned [*n* = 2 genomes]) were annotated as intact, incomplete, and questionable by Phaster. For two genomes representing serovars Lattenkamp and Poano, the topology of the core SNP phylogeny indicated that these two genomes do not belong to one of the major clades (A, B, C, D), so these genomes were categorized as “clade unassigned.” (**A**) Percent of the genomic content annotated as prophage regions by phylogenetic clade for 242 *Salmonella enterica* genomes. The percents were calculated using the total length (in base pairs) annotated as prophage by Phaster and the total base pairs in the genome. (**B**) Box plots displaying counts of the number of total, intact, incomplete, and questionable prophage regions among all *S. enterica* subsp. *enterica* genomes.

The genomic content of many bacterial pathogens has been shown to be impacted by horizontal gene transfer driven in part by lysogenic phages (13, 18–20). To determine how prophages may have shaped the resulting genomes of the foodborne pathogen *S. enterica*, we first characterized the prophage content of 242 *S*. *enterica* subspecies *enterica* genomes representing 217 serovars that are associated with human and animal clinical infections in the United States (21). On average, 3.9% of the genome was annotated as prophage regions (range: 0.4%–8.8%), which was consistent across the four major subsp. *enterica* phylogenetic clades, A–D and for the two isolates representing serovars Poano and Lattenkamp (*P* = 0.9, Kruskal–Wallis; Fig. 1A), which could not be assigned to a major phylogenetic clade. All 242 genomes were annotated as having at least one prophage region, with a total of 1,918 prophage regions identified.

**Fig 2:**
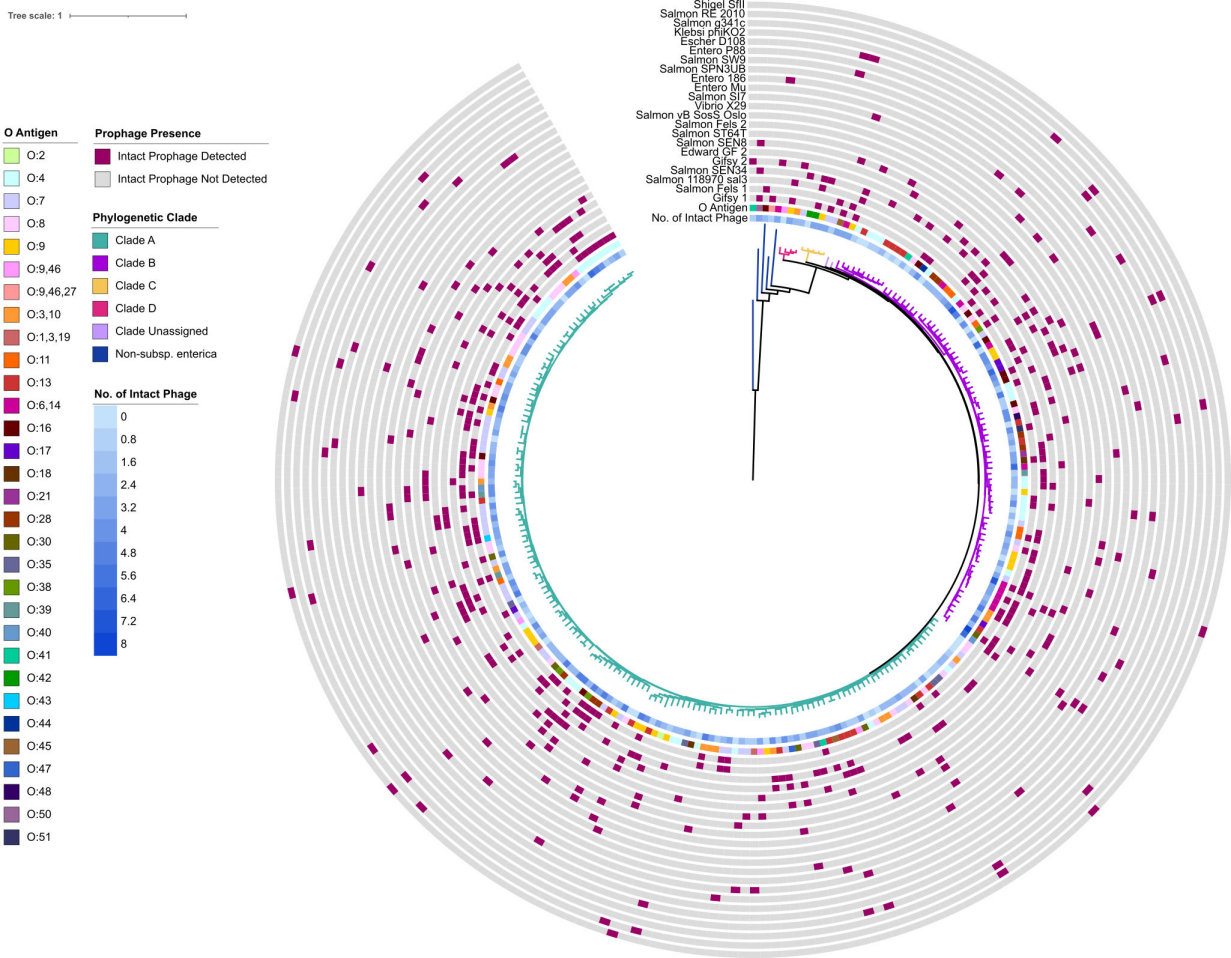
Distribution of intact prophage regions identified from *Salmonella* spp. genomes. A maximum likelihood phylogeny was inferred from core SNPs identified with kSNP3 for genomic assemblies representing 242 *S*. *enterica* subsp. *enterica* genomes and five additional assemblies representing subspecies *salamae* (II), *arizonae* (IIIa), *diarizonae* (IIIb), *houtenae* (IV), and *indica* (VI). Tree branches are colored to reflect the genome’s clade, which was assigned based on the tree topology and bootstrap support. The number of intact prophage regions identified with Phaster are indicated as the innermost line, with darker squares indicating higher numbers of intact prophage. The isolate O Group is indicated with a colored strip. For prophages with counts above five (i.e., present in more than five subsp. *enterica* genomes), presence (pink box) or absence (gray box) is shown.

Annotation with Phaster also provided predictions about the completeness of the prophage region, including whether a prophage is predicted to be intact, incomplete, or questionable (Fig. 1B) (22). Not surprisingly, the majority of the prophage regions were annotated as incomplete (987 regions; 51.5%), followed by regions that were predicted to be intact (569 regions, 29.7%) or questionable (362 regions; 18.9%). We chose to focus our analysis on the 569 regions that were considered to be “intact” as we hypothesized that these may be the most likely regions that could be excised and transferred to a new host. Among these 242 genomes, 206 (85.1%) contained at least one intact prophage region (average: two intact prophages; range: 0–8). Additionally, these 206 genomes represented all major phylogenetic clades previously characterized in *Salmonella* (4), suggesting that our data set includes diverse representation of potentially mobilizable prophage regions throughout *S. enterica* subsp. *enterica*. Among these intact prophages, 83 unique prophages (based on the phage name from blast identification included in Phaster output) were identified, with 22 of those prophages being detected in more than five genomes (representing 74.6% of all intact prophages), defined here as common prophages (Fig. 2). Gifsy 1 and Salmon Fels 1 (both predicted to be intact in 67 genomes) represented the most common intact prophages in *S. enterica* subsp. *enterica*. Mapping the presence of these prophages onto the *S. enterica* phylogeny revealed a sporadic distribution by phylogenetic clade (Fig. 2; Data Set S1), suggesting a complex pattern of evolutionary events accompanying the acquisition, maintenance, and loss of these prophages.

### Over half of the most common intact prophages identified in *S. enterica* subsp. *enterica* are also found in at least one other non-*enterica* subspecies

To better understand the distribution of these prophages across the *Salmonella* genus, we next examined their presence in an additional 61 genomes representing *S. bongori* (31 genomes) and representative genomes for the remaining *S. enterica* subspecies (*salamae*, *arizonae*, *diarizonae*, *houtenae*, *indica*, and *londinensis* [subsp. VII]*,* five genomes per subspecies). Similar to what we observed for the subsp. *enterica* genomes, within these non-subsp. *enterica* genomes, an average of 3.1% of genomic content was annotated as prophage (range: 0.1–8.1%, Fig S1A). Among 380 total prophage regions identified (average: 6, range: 1–13 prophage regions per genome), 110 (28.9%; Fig S1B) were predicted to be intact with phage Salmon 118970 sal3 (detected in 14 genomes) being the most common of the 31 unique prophages identified in the non-subsp. *enterica* genomes. Overall, 15 of the 22 common intact prophages in subsp. *enterica* were also found to be intact in non-subsp. *enterica* genomes represented here, demonstrating that many of these prophages are broadly present across the *Salmonella* genus.

### No significant associations were found between common prophages and O antigen groups

**Fig 3:**
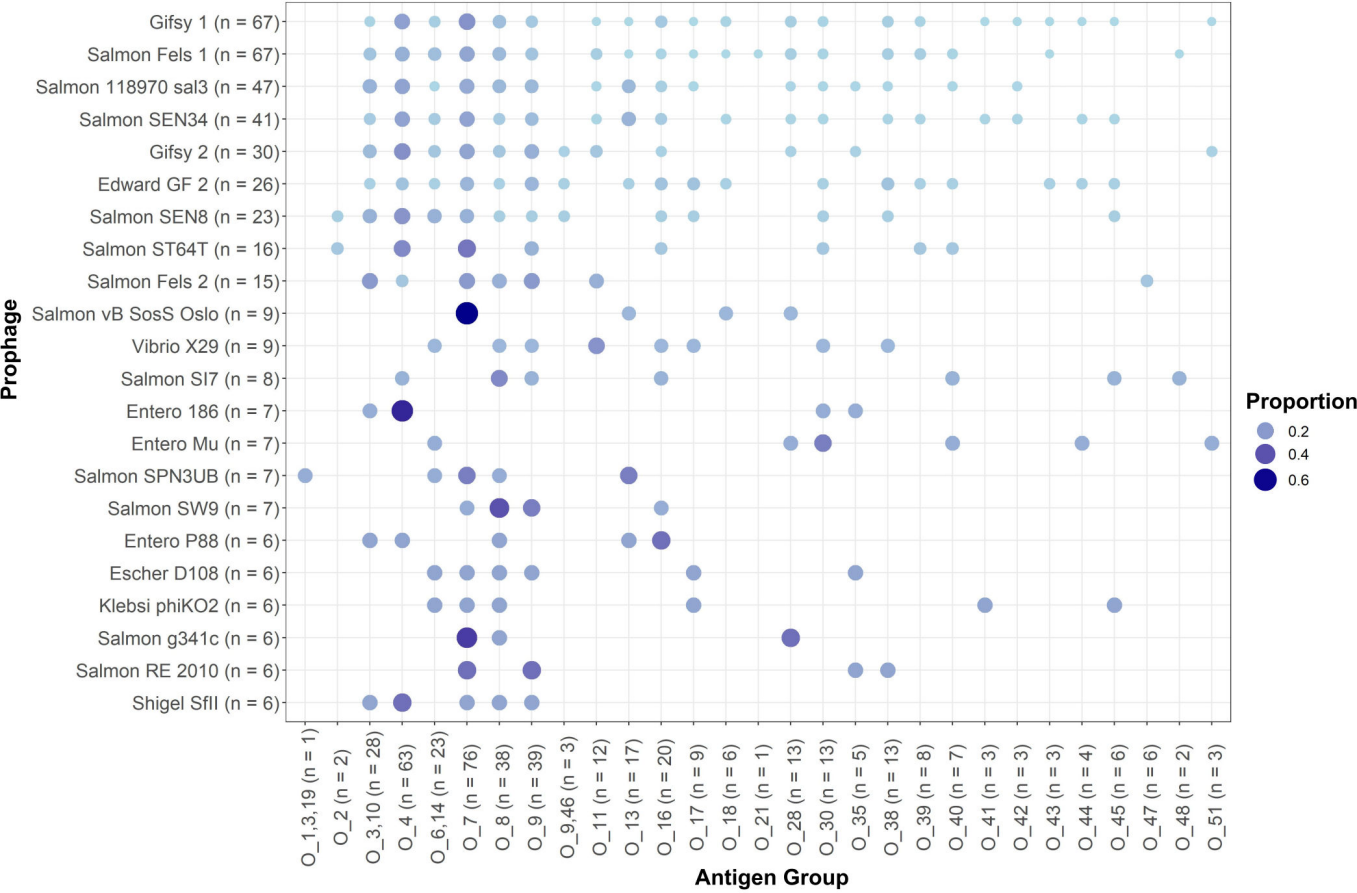
Distribution of the intact prophage regions across *Salmonella* serogroups. The bubble plot shows the number of prophages (*y*-axis; for prophages present in more than five *Salmonella* genomes) with *S. enterica* subsp. *enterica* genomes categorized by the O antigen group of the isolate (*x*-axis; number reflects the number of isolates in the serogroup included in the data set), represented as colored circles. The diameter and shading of the circle reflect the proportion of isolates in a given serogroup that were annotated as having an intact prophage region for the given prophage (*y*-axis), with larger, darker circles representing serogroups that had a high proportion of isolates with a given prophage.

The O antigen (terminal portion of LPS) has been demonstrated previously to be a receptor for multiple bacteriophages that infect *Salmonella* (23). Given the apparent sporadic distribution of the intact prophages (Fig. 2) and the previous observation that O groups evolved many times throughout the *Salmonella’*s evolutionary history (24), we next determined if there were any associations between O groups and the presence of specific prophages. Subspecies *enterica* genomes harboring intact prophages represented 29 unique O groups (average: 7; range: 1–36 genomes per O group; Fig. 3). Comparison of the presence/absence pattern of prophages by O group suggested that the distribution of most of these common prophages did not have a strong correlation with specific O groups (Fig. 3). Indeed, the proportion of genomes in each O group having a specific intact prophage did not vary significantly (Fisher’s exact tests; for *P*-values see Data Set S2), although for some O groups, the proportion of genomes having a specific prophage was notably higher (e.g., 67% of genomes in Group O:7 had intact Salmon vB Sos Oslo, and 57% of genomes in Group O:4 had intact Entero 186), suggesting that there may be a relationship between the presence of these prophages and isolates belonging to the O:4 and O:7 serogroups. Overall, this suggests that among the common intact prophages identified in subsp. *enterica* genomes, most are not associated with a specific O group.

### Intact prophage regions harbor coding sequences with a diverse range of predicted functions

**Fig 4:**
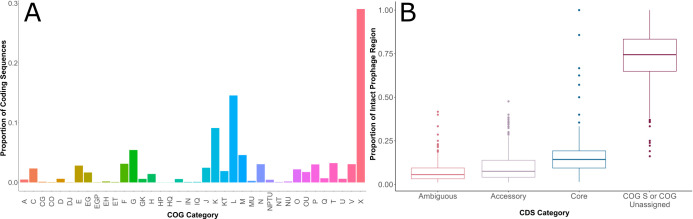
Classifications of CDSs from 569 intact prophage regions within 206 *S*. *enterica* subsp. *enterica* genomes. (**A**) Proportion of CDS assigned to each COG category for all phylogenetic clades for the 5,561 CDSs in the curated data set; the total number of genomes included in this analysis (206 genomes) represents the number of genomes from which intact prophage regions were identified with Phaster. COG annotations were determined using EggNOG. Data for COG categories “S” (Function Unknown) and “-” (No COG Category Assigned) are not shown as these COGs were not included in the curated CDS data set; raw data for this analysis is included in Data Set S2. COG categories are as follows: RNA Processing (A), Energy Production (C), Energy Production/Carbohydrate Metabolism (CG), Energy Production/Posttranslational Modification (CO), Cell Division (D), Cell Division/Translation (DJ), Amino Acid Metabolism (E), Amino Acid Metabolism/Carbohydrate Metabolism (EG), Amino Acid Metabolism/Carbohydrate Metabolism/Inorganic Ion Metabolism (EGP), Amino Acid Metabolism/Coenzyme Metabolism (EH), Amino Acid Metabolism/Signal Transduction (ET), Nucleotide Metabolism (F), Carbohydrate Metabolism (G), Carbohydrate Metabolism/Transcription (GK), Coenzyme Metabolism (H), Coenzyme Metabolism/Inorganic Ion Metabolism (HP), Coenzyme Metabolism/Secondary Metabolite Metabolism (HQ), Lipid Metabolism (I), Lipid Metabolism/Cell Motility (IN), Lipid Metabolism/Secondary Metabolite Metabolism (IQ), Translation (J), Transcription (K), Transcription/Signal Transduction (KT), Replication (L), Cell Wall/Membrane Biogenesis (M), Cell Wall/Membrane Biogenesis/Intracellular Trafficking (MU), Cell Motility (N), Cell Motility/Inorg Ion Met/Signal Transduction/Intracell Trafficking (NPTU), Cell Motility/Signal Transduction (NT), Cell Motility/Intracellular Trafficking (NU), Posttranslational Modification (O), Posttranslational Modification/Intracellular Trafficking (OU), Inorganic Ion Metabolism (P), Secondary Metabolite Metabolism (Q), Signal Transduction (T), Intracellular Trafficking (U), Defense Mechanisms (V), Mobilome (X). (**B**) CDSs were categorized as ambiguous, accessory, core, or COG S or COG Unassigned based on their predicted function by EggNOG. All 21,687 CDSs from 569 intact prophages were analyzed to determine the proportion of each CDS category by region. Classifications were based on EggNOG annotations and manual curation as defined in the methods.

During prophage excision, it is possible for bacterial host DNA to be excised and packaged into phage capsids, enabling potential horizontal transfer of cargo to a new host upon infection. To systematically identify and categorize CDSs that could provide some accessory function to the host *Salmonella* (see Materials and Methods for details; see Data Set S3 for exact terms), we first categorized intact prophage regions into clusters of orthologous groups (COGs) using EggNOG-mapper v2 (25). EggNOG annotated a total of 21,687 CDSs among the 569 intact prophages from 206 subsp. *enterica* genomes. After removing CDSs annotated as “Unknown Function” (COG S; *n* = 12,111) and “COG unassigned” (COG “-”; *n* = 4,015), the curated data set contained 5,561 remaining CDS annotations. Overall, these 5,561 CDSs were assigned to 38 unique COG categories, including those that represent a combination of single category annotations (i.e., COG EG encompasses functions related to Amino Acid Metabolism [E] and Carbohydrate Metabolism [G]); Fig. 4A). COG categories Mobilome (COG X); Replication, Recombination, and Repair (COG L); and Transcription (COG K) had the highest proportion of CDSs assigned, which was consistent for all clades, except Clade D (Fig. S2). Carbohydrate Transport and Metabolism (G) and Cell Wall/Membrane/Envelope Biogenesis (M) were also among the COG categories with the highest number of CDSs assigned suggesting that prophage cargo may play an important role in carbohydrate metabolism and cell wall modifications.

While the majority of CDSs within a given intact prophage could not be assigned a definitive function, 7.5% of CDSs on intact prophage regions (median; range: 1.1%–47.7%) were assigned a function that was considered to be “accessory” to the prophage (see Materials and Methods for a discussion of how these were assigned), which we hypothesized included some CDSs that could potentially provide a functional benefit to the host bacterial cell (Fig. 4B). These 1,661 accessory CDSs (representing 305 unique EggNOG definition terms) could be broadly categorized into (i) metabolic; (ii) cell surface structures; (iii) DNA replication, repair, and regulation; and (iv) virulence-associated functions. While CDSs were classified as accessory if their annotated function was not explicitly related to phage structural proteins or integration, some of these CDSs, especially those involved in DNA replication, repair, and regulation, could be part of the prophage replication module. Based on the distribution of these accessory CDSs, 73 were common (i.e., found in at least five different prophages or genomes; Data Set S3), which we then characterized further.

### Two-thirds of accessory CDSs on intact prophage regions are associated with replication, transcription, or metabolism

**Fig 5:**
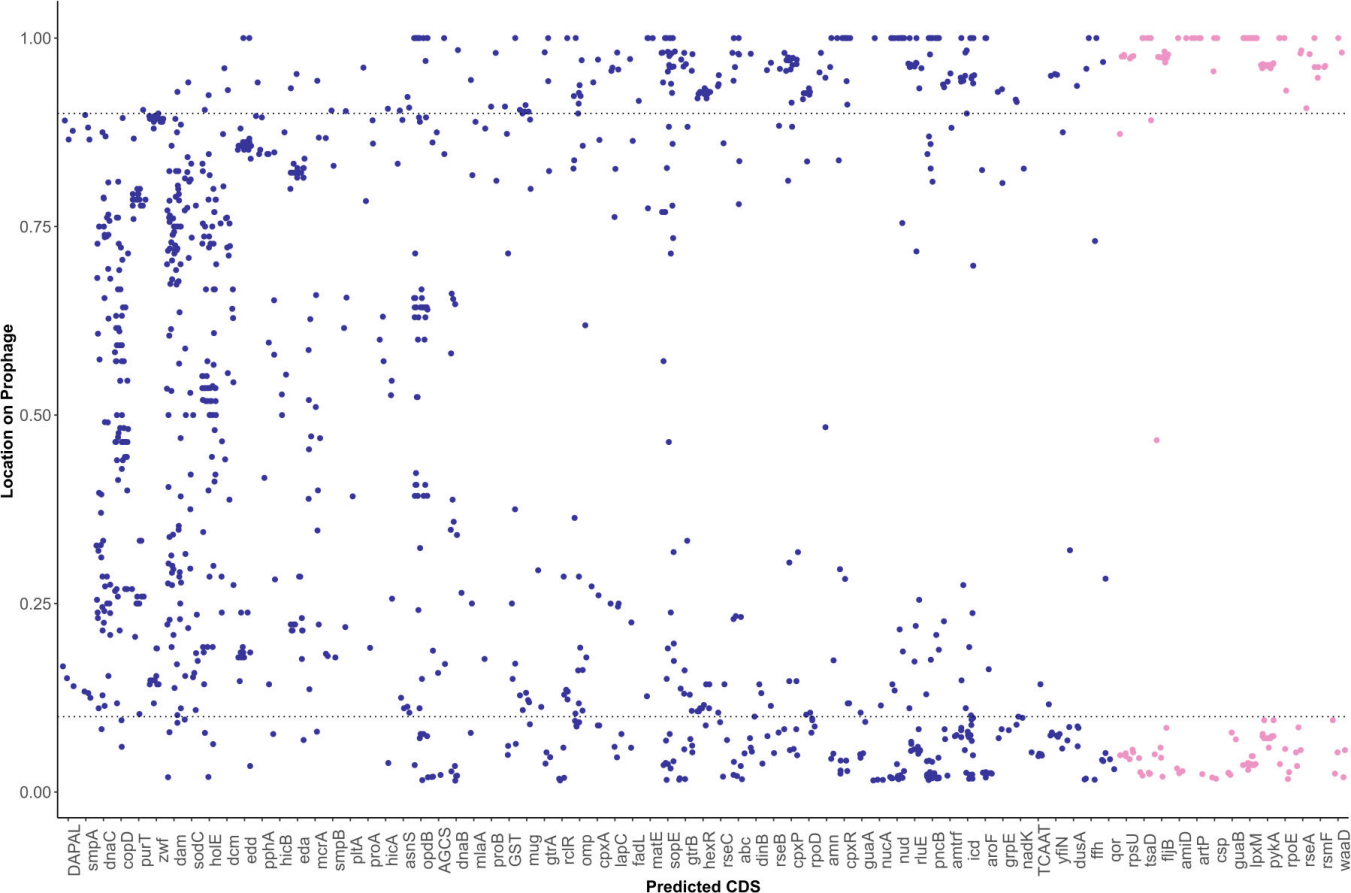
Position of accessory coding sequences on intact prophage regions. The position of the most common (presence in five or more intact prophage regions) accessory CDSs on intact prophages was calculated as a proportion where 0.0 indicates the first coding sequence annotated from the prophage, and 1.0 indicates the last coding sequence from that prophage (see Materials and Methods). Pink circles represent predicted CDS types where over 90% of the annotations were found in either the first or last 10% of the prophage; blue circles represent predicted CDS types with under 90% annotations found at either end of the prophage.

Not surprisingly, 26 of the 73 most common accessory CDSs had an annotated function related to DNA replication and repair (e.g., DNA polymerase θ subunit, endonucleases), transcription (e.g., sigma factors, transcriptional regulators, methyltransferases), and translation (e.g., tRNA synthetase); six of these CDSs consistently mapped to the ends of prophage regions (Fig. 5; see Materials and Methods for details about how this was determined). Metabolism-associated CDSs were the second most populous category, accounting for 22 of the 73 most commonly occurring accessory CDSs on intact prophages (Data Set S3); only two of these CDSs were located on the ends of prophage regions. These CDSs were associated with metabolism of a wide variety of compounds including carbohydrates (glucose-6-phosphate), amino acids, nucleotides, cofactors, and metal ions (Data Set S3). CDSs for carbohydrate metabolism included isocitrate dehydrogenase (*icd*) and genes associated with glucose-6-phosphate oxidation, while those for nucleotide metabolism encoded enzymes for purine biogenesis (*purT*, *proAB*, and *guaA*). CDSs related to siderophore synthesis and export (*iroC*, *fepBCDG*, and *entABCEHS*), zinc transport (*znuABC*), and copper resistance protein CopD were also identified (Fig. 5; Data Set S3). These data suggest that the majority of accessory CDSs on prophages facilitate DNA replication and repair and metabolism of a variety of compounds.

### Intact prophages carry CDSs associated with modification of LPS and cell surface proteins

Prophages have been previously shown to carry genes encoding proteins that modify the host cell to prevent infection with other bacteriophages that could use the same surface receptor (26, 27). Fifteen unique CDSs associated with functions related to modification of cellular surface structures were identified in the most commonly occurring 73 accessory CDSs, with five consistently occurring at the ends of the region (Fig. 5; Data Set S3). *fljB* encoding the phase 2 flagellin was almost exclusively found at the end of prophage regions identified as Salmon SEN 8 and Salmon Fels 2, suggesting either horizontal transfer of this locus or integration of these prophages adjacent to *fljB* in multiple serovars. CDSs for outer membrane porins (detected in 25 prophage regions) were also prominent among cell structure-associated CDSs. The GTR glucosyltransferase operon *gtrABC* was found intact in five prophage regions (Salmon ST160, Salmon ST64T, and Entero UAB Phi20), or as an incomplete operon in an additional five intact prophage regions (Data Set S3) (28). Also identified were CDSs associated with maintenance of the bacterial outer membrane, including those involved in outer membrane lipid asymmetry and the synthesis of lipid A (*waaD* and *lpxM*), but these were also almost exclusively found at the ends of prophage regions (Fig. 5). These results highlight that prophages carry a variety of CDSs associated with cell surface modification, with some having a predicted function related to modification of LPS (O antigen), potentially altering the serotype of the lysogenized cell.

### *sopE* and *sodC* are the predominant virulence-associated CDSs carried on intact prophages

Our analysis also identified CDSs that were found previously to play a role in virulence, namely, *sopE* and *sodC. sopE*, encoding a guanine nucleotide exchange factor, was identified on 44 different intact regions representing nine different prophages, while *sodC* was found on three different intact prophages from 29 subsp. *enterica* genomes, most commonly on regions annotated as Gifsy 2 (*n* = 22; 75.9%) but also on regions annotated as Gifsy 1 and Salmon Fels 1 (Data Set S3). *sodA*, encoding a superoxide dismutase with a function related to that of SodC (29), was detected on two different prophages (Yersin L 413C and Entero fiAA91 ss), neither of which was annotated as having *sodC*. The ADP-ribosylating toxin subunit encoded by *artA* or *pltA* was identified in five genomes on three different prophages (Salmon 118970 sal 3, Vibrio Χ29, and Gifsy 1; Data Set S3). Overall, these results suggest that genes encoding superoxide dismutases (SodC and, to a lesser extent, SodA) and SopE are the predominant virulence factors carried by intact prophages in *Salmonella*, although ArtAB and typhoid toxins can be horizontally acquired due to lysogenization with prophages.

### While some intact prophages carry antimicrobial resistance genes, fewer than 3% do

**Fig 6:**
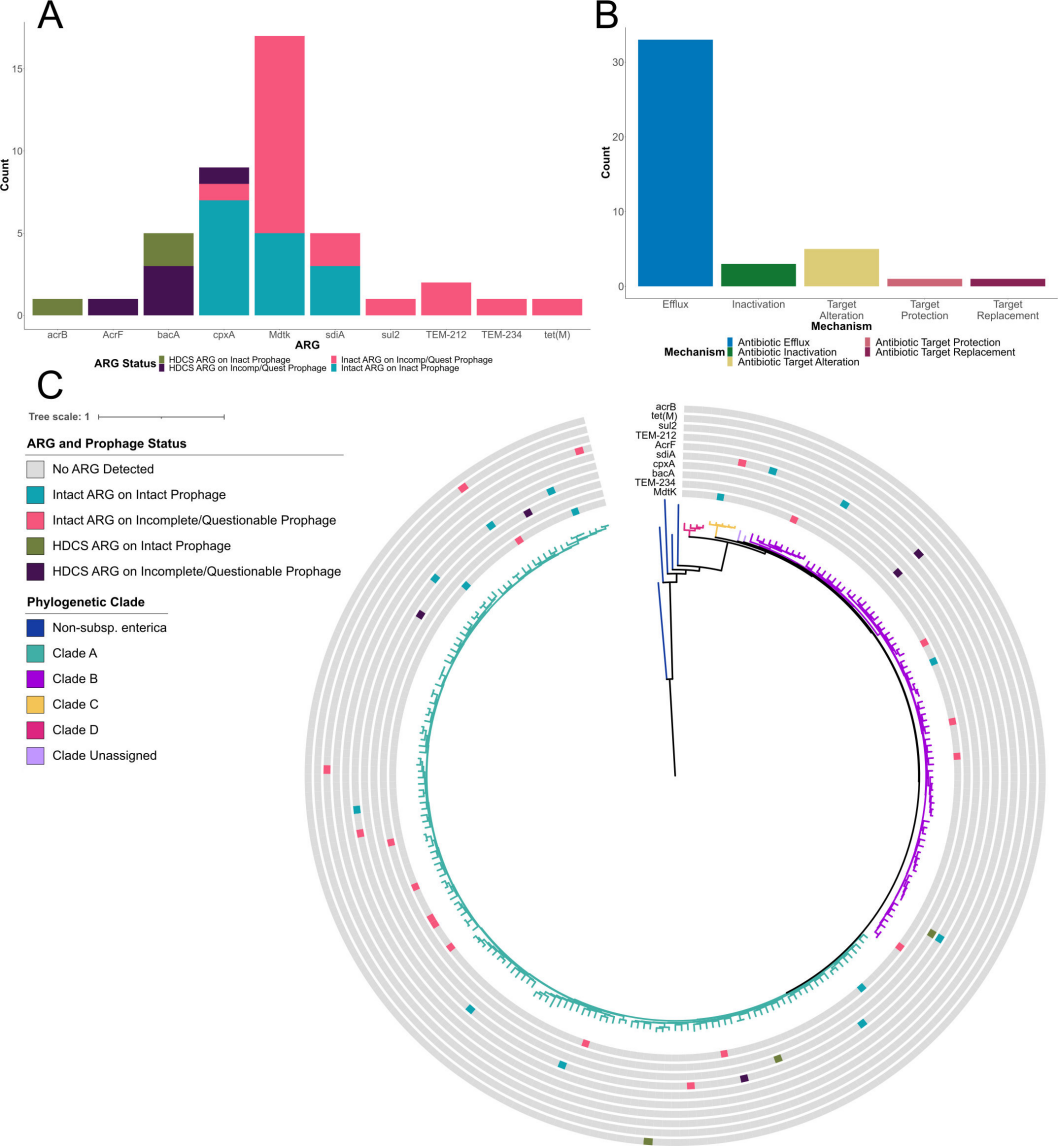
Analysis of ARGs found on intact, incomplete, and questionable prophage regions. The CARD database was used to identify and characterize ARGs (*n* = 43) on all prophage regions for the 242 *S*. *enterica* genomes. (**A**) Stacked bar plot shows the counts for the 10 unique AMR types identified. Colors indicate the status of the ARG (intact vs hypothetically disrupted coding sequence) and prophage (intact, incomplete, or questionable). (**B**) Bar plot shows all 43 ARGs (including those found on non-intact prophage regions) categorized by antimicrobial mechanism type. (**C**) Presence/absence distribution of ARG (colored [presence] or gray [absence] boxes) mapped onto the phylogenetic tree described in Fig. 2. ARGs are colored according to their status (intact or HDCS) and status of the prophage region they were found on (intact or incomplete/questionable).

Previous studies reported the presence of antimicrobial resistance genes (ARGs) on prophages, although the role of prophages in facilitating their transmission is contested (30, 31). EggNOG classification suggested the presence of three types of ARGs found on intact prophage regions: *acrB*, *mdtK*, and *bacA*, which were found within COG V (Defense Mechanisms). To more definitively characterize these and other ARGs, we executed a local blastn search to identify and extract ARGs on all prophage regions using reference sequences from the Comprehensive Antibiotic Resistance Database (CARD) (32). A total of 18 ARGs, including three that were predicted to be hypothetically disrupted coding sequences (HDCSs), were identified on intact prophage regions in subsp. *enterica* genomes with 25 additional ARGs detected on incomplete or questionable prophage regions (Fig. 6A). The 15 intact ARGs on intact prophage regions encompassed three unique ARGs and one antimicrobial resistance mechanism type (Fig. 6B). Unique ARGs that were found more than once were not associated with a specific prophage, with the exception of TEM-212 where both observations were found on the same prophage within the same genome (Table 1), suggesting that, in most cases, different prophages transfer different ARGs. The distribution of the ARGs was independent of the phylogenetic clade of the genome (Fig. 6C), and none were identified in genomes from Clade D (*n* = 4) or Clade Unassigned (*n* = 2), though this may be a result of the smaller number of genomes included to represent these phylogenetic groups. Therefore, while prophages can carry ARGs, intact ARGs on intact prophage make up a small percentage (0.9%, *n* = 15) of accessory cargo, suggesting that prophage-mediated acquisition of ARGs may not be the primary mode of recent acquisition for *Salmonella*.

**TABLE 1:** ARGs detected on intact, incomplete, and questionable prophage regions

ARG mechanism	CARD short name^*a*^	Count^*b*^	Proportion intact ARGs^*c*^	Associated prophages
Antibiotic efflux	MdtK	17	1	Salmon 118970 sal3, Cronob ENT39118, Gifsy 1, Clostr PhiS63, Edward GF 2, Erwini EtG, Lactob phiAQ113, Salmon vB SosS Oslo, Shigel Sf6
	cpxA	9	0.88	Yersin L 413C, Entero fiAA91 ss, Entero Wphi, Escher P2
	sdiA	5	1	Salmon SEN34, Entero mEp390, Entero SfV, Salmon Fels 2
	AcrF	1	0	Entero mEpX1
	acrB	1	0	Pseudo PaMx74
Antibiotic inactivation	TEM-234	1	1	Salmon SJ46
	TEM-212	2	1	Salmon SJ46
Antibiotic target alteration	bacA	5	0	Cronob ESSI 2, Entero 186, Escher 500465 1
Antibiotic target protection	tet(M)	1	1	Escher 500465 2
Antibiotic target replacement	sul2	1	1	Escher SH2026Stx1

^*a*^ Comprehensive Antibiotic Resistance Database (32).

^*b*^ Total count of intact and HDCS ARGs identified by CARD on intact and non-intact prophage regions. Categories of ARGs include intact ARG on intact prophage (*n* = 15), HDCS ARG on intact prophage (*n* = 3), intact ARG on non-intact prophage (*n* = 20), and HDCS ARG on non-intact prophage (*n* = 5).

^*c*^ ARGS were classified as (i) intact if they did not have a premature stop codon and had a coverage >0.8 or (ii) hypothetically disrupted coding sequences if they had a premature stop codon or a coverage of <0.8. The number of intact ARGs detected for each antibiotic resistance ontology term was divided by the total number of ARGs detected for that term.

## DISCUSSION

Mobile genetic elements, such as prophages and plasmids, are key mediators of horizontal gene transfer in bacteria (18, 33). Recent characterizations of prophage regions in diverse serovars have offered insight into the evolutionary history of *Salmonella* and have identified the potential role of prophage cargo in mediating *Salmonella*’s interactions with its many hosts (13, 14, 34). The work presented here demonstrates that prophage regions across *Salmonella* spp. are considerably diverse and facilitate the transfer of CDSs associated with a variety of functions. While we identified a number of CDSs representing traditional virulence factors (SodC, SodA, and SopE), our analyses also identified a number of CDSs with a predicted function related to cell surface structure modification, heavy metal resistance, and metabolism of a variety of compounds, which may play an important role in facilitating *Salmonella*’s host–pathogen interactions.

### Many intact prophages are broadly distributed throughout the *Salmonella* genus

Previous comparative genomic studies identified differences in prophage content as one of the key differences between clades within a serovar (14, 35, 36). Nearly one-third of the prophages found within our *S. enterica* subsp. *enterica* genomes were also found among genomes from *S. bongori* and *S. enterica* subsp. *salamae*, *arizonae*, *diarizonae*, *houtenae*, *indica*, and *londinensis*. While serovars of *S. enterica* subsp. *enterica* cause roughly 99% of human salmonellosis (37), analyses focused on *S. bongori* and other *S. enterica* non-*enterica* subsp. will be beneficial for understanding the evolution of *Salmonella*’s prophage repertoire and frequency of mobilization of different prophages, and, more importantly, the cargo that they carry.

### Prophages carry cargo with a diverse range of functions

Prophages are known to facilitate acquisition of toxins in many pathogenic bacteria, including the Shiga toxin encoded by Shiga toxin-producing *Escherichia coli* (STEC), botulinum neurotoxins BoNTX in *Clostridium botulinum*, and the cholera toxin in *Vibrio cholerae* (38–40). Previous comparative genomic studies of prophages in *Salmonella* established that prophages can facilitate the transfer of virulence factors, with the most well-studied examples being *sodC*, encoding a superoxide dismutase, and *sopE*, encoding a guanine nucleotide exchange factor associated with membrane ruffling in infected eukaryotic cells (16, 41). In our data set, we identified *sopE* and *sodC* on intact prophage regions in 39 and 29 subsp. *enterica* genomes, respectively. This further demonstrates that *sopE* and *sodC* can be carried on multiple prophages and are found broadly across subsp. *enterica* lineages. We also identified ArtAB toxin and one instance of typhoid toxin being found on different intact prophages in five serovars from subsp. *enterica* clades A and B, confirming that non-DT104 lineages can harbor prophages encoding *artAB* as well (42–44). A recent analysis of ArtB toxin subtypes suggested that *artB* is encoded on a variety of prophages (45). Together, these findings continue to challenge our understanding of the potential role of phage-encoded toxins in *Salmonella*’s pathogenesis and virulence potential.

In addition to these traditional virulence factors, we also identified a number of CDSs that may facilitate metabolic adaptations that could provide some fitness benefit allowing *Salmonella* to propagate in a new host or environment. Previous studies demonstrated that CDSs within prophage regions can affect host metabolic activity, such as the *dgo* operon in facilitating D-galactonate metabolism and *pckA* involved in gluconeogenesis (13). The broad range of functional cargo identified here suggests that prophages in *Salmonella* may confer a wide variety of adaptations that might allow lysogenized strains to persist or survive in different environmental conditions.

### Prophages likely played an important role in the evolution of *Salmonella* surface antigens

Past studies have shown that prophages played an important role in facilitating horizontal acquisition of surface-modifying enzymes, such as glycosyltransferases (13, 46). Prophages are known to facilitate horizontal acquisition of CDSs that modify the O antigen, thereby preventing superinfection (infection with other phages that might use the same receptor) (47, 48). In our data set, we found that multiple unique intact prophages carry CDSs that are annotated as modifying the O antigen (e.g., *gtrABC*). Variation in the O antigen due to prophage-mediated acquisition of surface-modifying enzymes (e.g., glycosyltransferases, acetyltransferases) has an impact on the resulting O antigen structure. Indeed, lysogenization with P22 results in glucosylation of the galactose subunit of the basal O antigen, resulting in recognition with O1 antiserum (representing the “1” O antigen in the White–Kauffman–Le Minor serotyping scheme) (5, 28). The *gtrABC* operon is known to glycosylate different moieties on sugars present at the terminal end of the LPS (the “O antigen”). Analyses of the O antigen diversity suggest that *gtrABC* carried by prophages may account for the structural modifications of the O antigen in antigen group O:6,14 (48). Our results support that in addition to P22, other prophages identified by Phaster as Entero UAB Phi20, Salmon ST160, and Salmon ST64T encode *gtrABC* (49). The gain and loss of prophage cargo, or more specifically the cell surface-modifying cargo that they carry, could be one possible explanation for the emergence of so-called polyphyletic serovars, which has important implications for surveillance efforts that rely on serological typing (4, 50). Although our analyses did not identify any significant associations between the presence of intact prophages and specific O antigen groups, some prophages (such as Salmon vB SosS Oslo) do carry cargo that is involved in the synthesis of Kdo_2_-lipid A (51). Additional studies using larger numbers of genomes of representative isolates expressing the same serotype would be helpful in further identifying prophages whose cargo result in conversion to a different serotype.

Surprisingly, Phaster identified 20 CDSs for the flagellin FljB subunit (initially identified as FliC by EggNOG) on five different intact prophages; all but one of these CDSs mapped to the ends of the prophage region. While these results may suggest that prophages can carry the phase 2 flagella locus, it is also probable that the attachment site of these prophages is adjacent to the *fljAB-hin* locus. Indeed, Soyer et al. hypothesized that imprecise excision of a prophage adjacent to *fljAB* and *hin* led to the loss of the phase 2 antigen in several lineages of monophasic Typhimurium (also known as I 4,[5],12:i:-) (52). Transfer of *fliC* from an *S*. Montevideo strain into the ancestor of *S*. Mbandaka is hypothesized to have given rise to serovar Lubbock, although the authors did not specify whether this horizontal event was mediated by prophages (53). Together, our observation of the *fljAB-hin* locus being present on a prophage or directly adjacent to a prophage, and other reports of prophage excision potentially leading to the loss of this locus in monophasic serovars, suggests that prophages may also influence *Salmonella* flagellar antigens, although experimental data are needed to definitively assert this.

Our analyses suggest that prophages and their cargo contributed importantly to the diversification of *Salmonella* spp. Experimental analyses examining whether the common intact prophages identified here can indeed be excised will be helpful for understanding the transmission of the CDSs identified in this study and their potential role in facilitating *Salmonella*’s interactions with its hosts and environments. Evolutionary analyses estimating the gain and loss events that led to the distribution of prophages throughout *Salmonella* will enable a more complete understanding of the role that these phages played and continue to play in shaping *Salmonella’*s genetic diversity. Finally, analyses characterizing the conservation and loss of these prophages among genomes within a serotype or standardizing prophage nomenclature are needed, especially in light of emerging evidence of “hypervirulent” subtypes within a serotype and the potential use of virulence markers in serovar-independent risk assessments (9). Overall, through characterization of the diversity and distribution of prophages, the entangled role of prophages in driving the interactions and evolution of *Salmonella* is beginning to be unpacked.

## MATERIALS AND METHODS

### Genome selection criteria

Genomic assemblies for a previously reported set of 217 *S*. *enterica* subsp. *enterica* serovars, represented by 242 genomes, were analyzed in this study (Data Set S1) (21). Serovar selection criteria included (i) human case prevalence in the United States from 2006 to 2016, (ii) nonhuman clinical and animal non clinical case prevalence in 2012, and (iii) novel or rare clades of *S. enterica* subsp. *enterica* as outlined in a prior study (21). Genomes within these serovars were then selected based on prevalence of whole-genome sequence (WGS) data availability using the National Center for Biotechnology Information (NCBI) Pathogen Detection Database to identify the most prevalent single-nucleotide polymorphism (SNP) cluster (https://www.ncbi.nlm.nih.gov/pathogens/). For serovars that were previously described as paraphyletic or polyphyletic clades (4), a single genome was chosen per clade. Within each SNP cluster, genomes were selected based on their having (i) the highest *N*_50_ value among isolates in the SNP cluster, (ii) their being sequenced on an Illumina platform (required for the various pipelines used), and (iii) their having a genome size of 4.5–5.7 million base pairs (expected genome size for *Salmonella* spp. isolates). Additional genomes representing *S. enterica* subspecies *salamae*, *arizonae*, *diarizonae*, *houtenae*, and *indica* (one genome per subspecies) were included for inferring phylogenetic structure. Assemblies for selected genomes were downloaded from the NCBI Pathogen Detection Database; all genomes were assembled with SKESA version 2.2 (54) and annotated with PGAP version 0.2.0 (55). Serotype information was confirmed with the *Salmonella in silico* typing resource (SISTR) version 1.0.2 (56).

### Identification of core genome SNPs, inference of core genome phylogeny, and clade classification

Clade designations for *S. enterica* subsp. *enterica* genomes were assigned based on phylogenies inferred from core SNPs. kSNP version 3.1 with a kmer size of 19 was used to identify and align 13,926 core SNPs in all genomes (57). IQ-TREE v.2.0.7 was used to infer phylogenies using the model GTR + G + ASC (general time reversible with gamma distribution and ascertainment bias correction) and 1,000 ultra-fast (UF) bootstraps repetitions (58, 59). Clades were assigned letters A–D as defined previously by Worley et al. (4). For two serovars (Lattenkamp and Poano), the phylogeny inferred from core SNPs could not be used to definitively assign these genomes to a clade, and therefore, they are presented as “clade unassigned” in the data set as they potentially belong to a novel phylogenetic clade (4).

### Identification and characterization of prophage regions

Phaster (https://phaster.ca/) was used with default settings to identify, extract, and annotate prophage regions from all *Salmonella* spp. genomes (22, 60). Phaster uses three levels (“intact,” “incomplete,” and “questionable”) to rate the completeness of the prophage region that it identifies (22, 60). The output summary annotated prophage regions as intact, incomplete, or questionable and included the common phage name, region length, total protein number and phage hit protein number. The common intact prophages (defined here as presence in >5 subsp. *enterica* genomes) were identified using the phage name assigned by Phaster, which is based on blast scores obtained by comparing the prophage region to a database of bacteriophage genomes. Prophages were counted by their presence in the genome, not by number of regions, due to there being 19 genomes that each contained two regions annotated as the same prophage (i.e., two regions annotated as Gifsy 1 in the same assembly).

### Characterization of prophages from non-subspecies *enterica* genomes

Assemblies for 61 genomes representing *S. bongori* and *S. enterica* subsp. II, IIIa, IIIb, IV, VI, and VII were included (for accession numbers see Data Set S1). All available *S. bongori* assemblies available on NCBI (*n* = 32; date accessed, 15 July 2021) were downloaded. One assembly was excluded due to poor sequence quality (GCA_003452365.1, *n* = 446 contigs), and therefore, a total of 31 assemblies representing 10 unique serotypes were included in the final data set. Representative assemblies for the remaining *S. enterica* subspecies were selected based on the number of isolates in each NCBI Pathogen Detection Database SNP cluster (date accessed, 15 July 2021). Assemblies with the highest *N*_50_ values from each of the top five most populous SNP clusters representing unique serotypes were selected for each subspecies (i.e., II, IIIa, IIIb, IV, and VI); for subspecies VII, due to the small number of assemblies available, selecting unique serotypes was not possible, and therefore, four of the five selected assemblies have an identical serotype. Prophage regions were characterized using Phaster following the same methods for the *S. enterica* subsp. *enterica* genomes, and the presence of the common prophages identified among intact *S. enterica* subsp. *enterica* genomes were compared in the non-subsp. *enterica* genomes.

### Characterization of coding sequences encoded on intact *S. enterica* subsp. *enterica* prophage regions

CDSs for intact prophage regions from *S. enterica* subsp. *enterica* were identified and annotated with Prokka (61). To assign a putative function, CDSs were then annotated using EggNOG-mapper v2 (http://eggnog-mapper.embl.de/) with percent identity, query coverage, and subject coverage set to 60% (25, 62); a threshold of 60% was determined based on results from a preliminary analysis using intact prophage regions from five randomly selected genomes in the data set (Data Set S3). EggNOG output for the 206 subsp. *enterica* genomes with intact prophage regions detected by Phaster was manually curated using the following steps (note that all raw data are available in Data Set S3). First, CDSs annotated as COGs “-” (representing CDSs with “no COG category assigned”) or “S” (representing CDSs with “Function Unknown“) were removed (*n* = 16,126 CDSs), producing the curated data set of 5,561 CDSs. Second, the unique “description” terms for each CDS within each COG were identified from EggNOG output within the curated data set. The “description” annotation was chosen for the primary analysis from the annotations provided due to its completeness across the curated data set. Third, individual CDSs were classified as “ambiguous,” “core,” or “accessory”; core CDSs were those with (i) COG definitions for COG X (mobilome), (ii) terms related to the prophage life cycle and maintenance consistent with those considered phage by Phaster (e.g., capsid, head, integrase, plate, tail, fiber, coat, terminase, portal, protease, and lysin), or (iii) were annotated as phage related by any EggNOG annotation (see Data Set S3 for full list of EggNOG definitions that we considered “core” CDSs) (22). The remaining CDSs were defined as “ambiguous” or “accessory” depending on their EggNOG annotations. Ambiguous CDSs contained EggNOG annotations where the functional role of the CDS was unclear due to the following: (i) more than two of the 13 annotation categories being incomplete, (ii) functional annotations being inconsistent, or (iii) CDSs being described as putative, hypothetical, locus tag, or domain of unknown function. All other CDSs were considered accessory CDSs (see Data Set S3 for a complete list of all CDSs and their assigned category). For *gtrC* (accession number NC_004348.1) and *sopE* (accession number AF153829.1), a blastn search was executed to confirm the identity and presence of these CDSs since they are known to be found on intact prophages but were not annotated as “GtrC or SopE” by EggNOG.

### Identification of COG X CDSs

The NCBI Database of COGs classifies prophage and transposon terms into COG X (Mobilome). A manual analysis was performed to determine the presence of any COG Definitions within the CDS data that were annotated as a different COG category by EggNOG. CDSs were considered COG X if the “eggNOG_Ogs” contained a COG X Definition as defined by NCBI (https://www.ncbi.nlm.nih.gov/research/cog/cogcategory/X/; date accessed, 10 September 2023). Of the 12,687 total CDSs annotated by EggNOG, 4,385 were defined as COG X. During the analysis of genetic content within prophage regions, any COG X terms were considered “core CDSs” (Data Set S3).

### Identification of putatively prophage adjacent CDSs

Phaster additionally searches all prophage regions for a CDS with an annotated function “integrase” for *attB* sites, which are short nucleotide repeats that the software predicts to be the attachment site where the phage genome integrated into the bacterial genome. Due to the methods implemented by Phaster to identify *attB* sites, we also reasoned that for some prophage genomes, it is possible that CDSs adjacent to the prophage regions could potentially be misclassified as being located on a prophage. The locations of CDSs within the curated data set belonging to the common 73 accessory CDSs were mapped using data from the EggNOG annotations of intact prophage regions identified by Phaster (22, 25). For each intact region, the total number of CDSs per region was identified, and each CDS was assigned a location in the region based on the query number from EggNOG output. For an individual CDS, the location (based on the query number) was divided by the total number of CDSs in the region. For each predicted CDS type, the number of CDSs with location values ≤0.1 or ≥0.9 (arbitrarily selected to represent the first 10% or last 10% of the prophage region) were divided by the total number of counts for the predicted CDS to get an overall location value. Predicted CDSs with an overall location ≥0.9 were considered to be “putatively prophage adjacent CDSs” as over 90% of the observations for that predicted CDS were in the first 10% or last 10% of the region (i.e., at the “ends” of region).

### Analysis of ARGs in prophage regions

All prophage regions identified in *S. enterica* subsp. *enterica* genomes were compared to CARD databases for the detection of ARGs using blastn (32). Genes were classified into putative functional (no premature stop codon and has a coverage >0.8) (intact), non-functional (premature stop codon or 0.3 < coverage < 0.8) (hypothetically disrupted coding sequences), and absence (no hits or coverage <0.3) (63). Hits were removed if they (i) were annotated as “absent,” or (ii) the gene coverage was not complete. In the case of duplicates, the hit with the best bitscore was selected.

### Data analysis and visualization

Data analysis and statistics were conducted using R Statistical Software version 4.1.2 (64). The tidyverse package version 2.0.0 and readxl package version 1.4.2 were used for data analysis (65, 66). The RVAideMemoire package version 0.9.83 was used for Fisher’s exact statistical tests (67). The ggplot2 package version 3.4.4 (68) and the Interactive Tree Of Life (iTOL) (69) were used for data visualization.

## Data Availability

Sequence data are publicly available in the NCBI database; see Data Set S1 for accession numbers. Data for this article can be accessed in the supplemental material (Data Sets S1 to S3).

## SUPPLEMENTAL MATERIAL

Data Set S1 msphere.00031-24-s0001.xlsx Isolate and prophage metadata.

Data Set S2 msphere.00031-24-s0002.xlsx Table of *P*-values for analysis of presence of specific prophages within O antigen groups.

Data Set S3 msphere.00031-24-s0003.xlsx Tables from analysis and classification of CDSs within intact regions.

Figure S1 msphere.00031-24-s0004.docx Summary of prophage regions among 303 *Salmonella* spp. genomes

Figure S2 msphere.00031-24-s0005.docx Proportion of COG categories for 5,561 CDSs by phylogenetic clade.

ASM does not own the copyrights to Supplemental Material that may be linked to, or accessed through, an article. The authors have granted ASM a non-exclusive, world-wide license to publish the Supplemental Material files. Please contact the corresponding author directly for reuse.
