# Supplementary material for: What’s on a prophage: analysis of Salmonella spp. prophages identifies a diverse range of cargo with multiple virulence- and metabolism-associated functions
Source: mSphere. 2024 May 22;9(6):e00031-24. doi: 10.1128/msphere.00031-24 (PMC11332146; doi:10.1128/msphere.00031-24)
Supplement: Figure S1 — Summary of prophage regions among 303 Salmonella spp. genomes [file msphere.00031-24-s0004.docx]

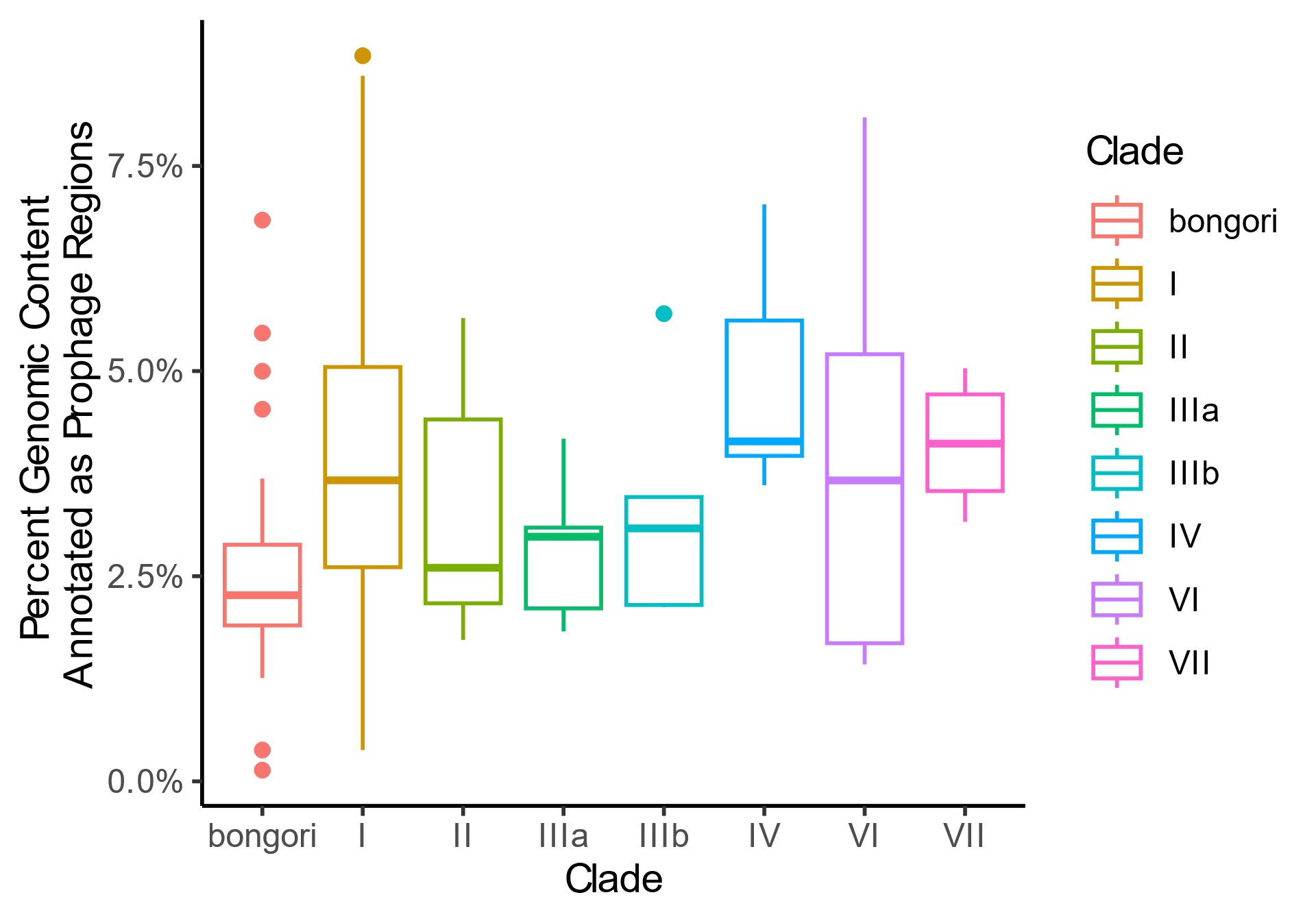


**A**

**B**

**
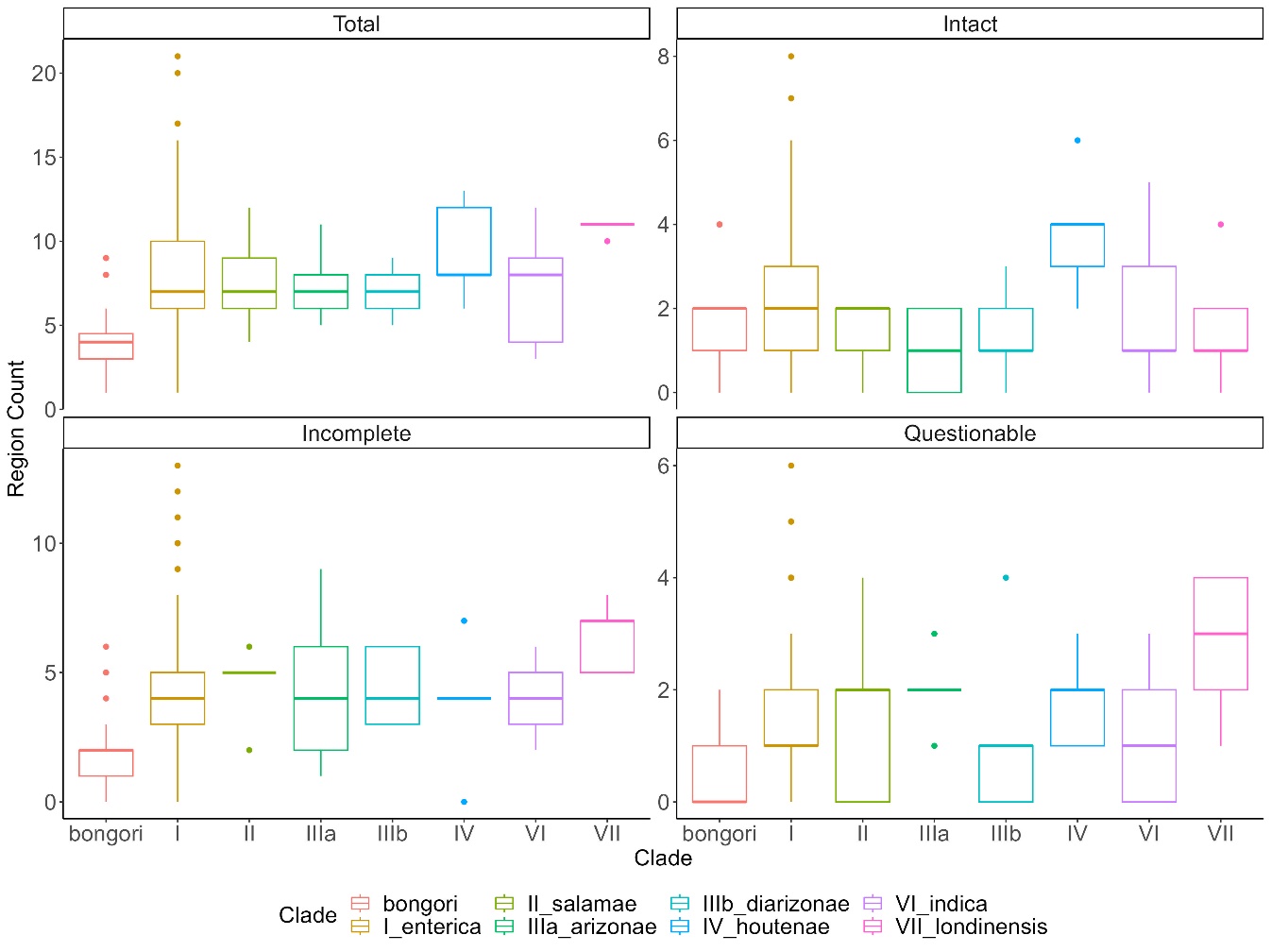
**

**Supplemental Figure 1.** Summary of prophage regions among 303 *Salmonella* spp. genomes. **(A)** The percent of genomic content annotated as prophage regions by Phaster using the total length in base pairs annotated as prophage and the total base pairs in the genome. **(B)** Prophage regions were annotated as intact, incomplete, or questionable by Phaster; “Total” shows all region types combined
