# Supplementary material for: What’s on a prophage: analysis of Salmonella spp. prophages identifies a diverse range of cargo with multiple virulence- and metabolism-associated functions
Source: mSphere. 2024 May 22;9(6):e00031-24. doi: 10.1128/msphere.00031-24 (PMC11332146; doi:10.1128/msphere.00031-24)
Supplement: Figure S2 — Proportion of COG categories for 5,561 CDSs by phylogenetic clade. [file msphere.00031-24-s0005.docx]

**
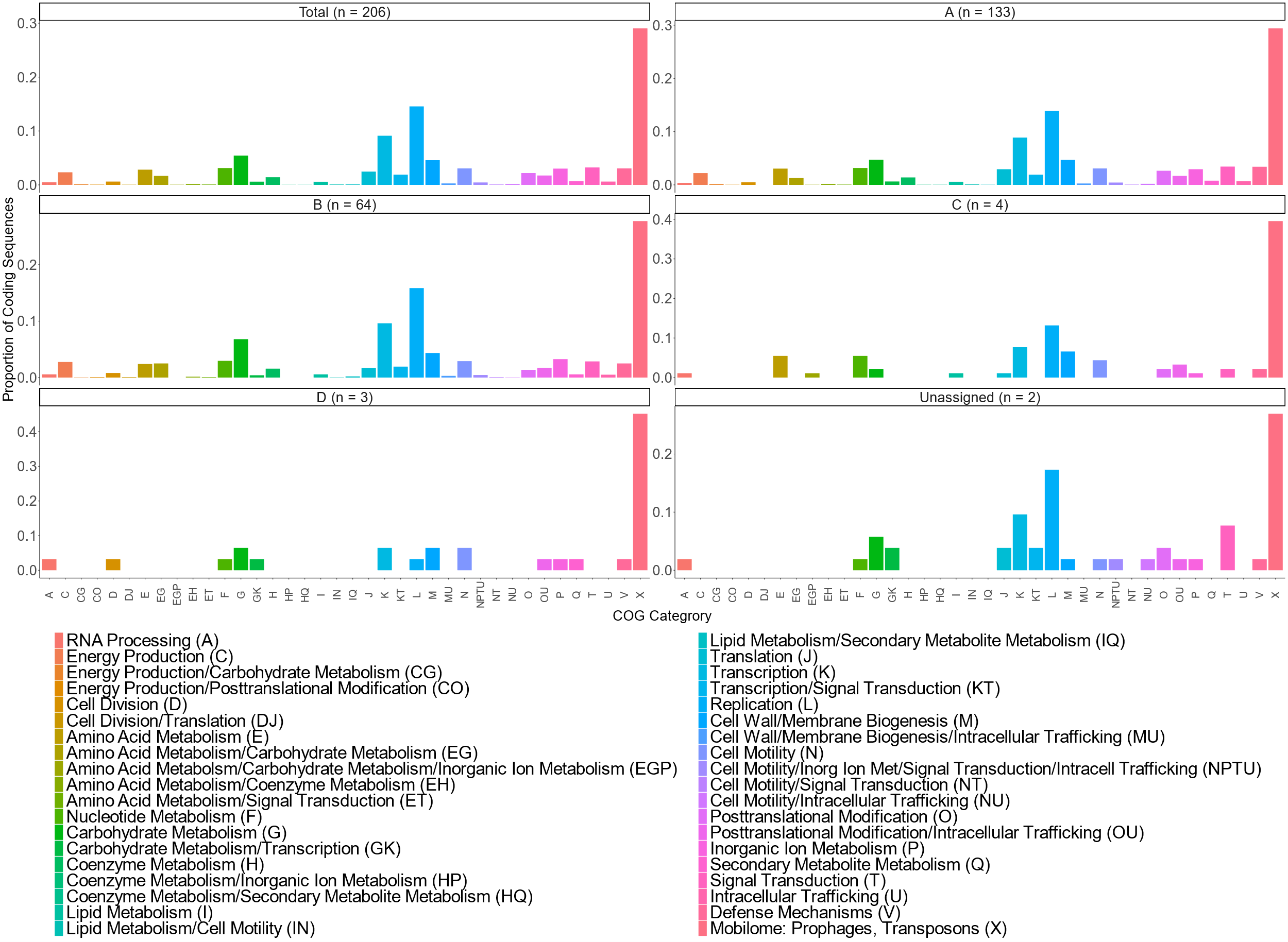
**

**Supplemental Figure 2.** Proportion of COG Categories for 5561 CDSs by phylogenetic clade. Proportions of CDSs annotated by EggNOG are shown for the 206 *S. enterica* subsp. *enterica* isolates with intact prophage regions. Numbers in parentheses indicate the number of isolates represented for each clade; total includes all clades combined. Data for COG S (Function Unknown) and COG “-” (No COG assigned) is not shown; full raw data is available in Supplemental Dataset 3.
